# Shedding Light on Penetration of Cereal Host Stomata by Wheat Stem Rust Using Improved Methodology

**DOI:** 10.1038/s41598-019-44280-6

**Published:** 2019-05-28

**Authors:** Shyam Solanki, Gazala Ameen, Pawel Borowicz, Robert S. Brueggeman

**Affiliations:** 1 Department of Plant Pathology, North Dakota State University, Fargo, ND 58108-6050 USA; 2 Department of Animal Sciences, North Dakota State University, Fargo, ND 58108-6050 USA

**Keywords:** Light responses, Virulence, Stomata

## Abstract

Asexual urediniospore infection of primary cereal hosts by *Puccinia graminis* f. sp. *tritici* (*Pgt*), the wheat stem rust pathogen, was considered biphasic. The first phase, spore germination and appressoria formation, requires a dark period and moisture. The second phase, host entry by the penetration peg originating from the appressoria formed over the guard cells, was thought to require light to induce natural stomata opening. Previous studies concluded that inhibition of colonization by the dark was due to lack of penetration through closed stomata. A sensitive WGA-Alexa Fluor 488 fungal staining, surface creation and biovolume analysis method was developed enabling visualization and quantification of fungal growth in planta at early infection stages surpassing visualization barriers using previous methods. The improved method was used to investigate infection processes of *Pgt* during stomata penetration and colonization in barley and wheat showing that penetration is light independent. Based on the visual growth and fungal biovolume analysis it was concluded that the differences in pathogen growth dynamics in both resistant and susceptible genotypes was due to light induced pathogen growth after penetration into the substomatal space. Thus, light induced plant or pathogen cues triggers pathogen growth *in-planta* post penetration.

## Introduction

The biotrophic fungal pathogen *Puccinia graminis* f. sp. *tritici* (*Pgt*) causes “Wheat Stem Rust”, which is a devastating disease of the cereal hosts wheat and barley; staple food crops produced in the world’s grain baskets^1–4^. The mechanisms of *Pgt* infection on their primary grass hosts requires asexual urediniospores landing on the stem or leaf surfaces under moist conditions conducive to spore germination, a process that also evolved to occur at night. After *Pgt* spore germination, the germ tubes grow perpendicular to leaf veins until they encounter stomata. The topology of specific host guard cells plays an important role in stomata identification and the induced formation of appressorium around 4–16 hours post inoculation^5^. The appressoria form a penetration peg and substomatal tube between the two guard cells to initiate host penetration, a mechanism that was previously determined to be light dependent^6^. Penetration is shortly followed by substomatal growth, which includes the formation of primary infection hyphae (PIH) that grow intracellularly until they encounter mesophyll cells. Contact with mesophyll cells induces differentiation and the formation of haustorial mother cells (HMC). From the HMCs located outside of the mesophyll cells degradation of the cell wall occurs followed by invagination of the host cell’s plasma membrane leading to the formation of intracellular haustoria. The haustoria are the pathogen’s feeding structures, which are the powerhouses of pathogen colony growth. The haustoria also facilitates pathogen manipulation of the host as the pathogen hijacks host cell physiology utilizing an effector repertoire that establishes an artificial nutrient sink that leads to pathogen feeding and profuse growth^7^.

An early study by Helen Hart^8^ using the wheat varieties Webster and Little club inoculated with *Pgt* after extended dark periods post inoculation showed that the majority of spores failed to penetrate wheat. However, the appearance of a few, minute pustules near the leaf tips were concluded to have possibly developed from pathogen entry through hydathodes or were due to forced entry. Inoculations that were made away from the leaf tips and given nine day extended dark periods did not resulted in any sporulation in the wheat lines except one case that was attributed to the possibility of a random open stomata. Henceforth, penetration through the stomata guard cells was considered to be light dependent for stem rust and it was concluded that appressoria remain quiescent on top of stomata until light induced stomatal opening facilitated pathogen penetration^5,6,9–11^.

The effect of light and CO_2_ concentration on stomata penetration by *Pgt* was also studied in wheat^6,9^. The researchers conclude that if a 20 hour extended dark period was provided after inoculation, then only 2–5% penetration occurred. However, if seedlings were exposed to a CO_2_ free 10-hour dark environment after incubation, the stem rust penetration greatly increased to 24.6–28.4%. In the presence of 5% CO_2_ for 10 hours in the light after dark incubation, stomata penetration was greatly reduced from ~60% to ~2.4%. Thus, this study concluded that plant stomata opening and stimulation by daylight was not the limiting factor for stem rust penetration, but instead CO_2_ concentration also plays a major role. It was further speculated^9^ that in the dark, due to respiration and the absence of photosynthesis, increased intercellular CO_2_ levels negatively impacted *Pgt* penetration through stomata. In another study, Burrage^12^ subjected wheat plants to different degrees of water stress in the presence of light after the initial dark and moist incubation period and showed that the infection percentage reduced to a level at par with dark treated plants. These findings suggested that other factors that manipulate stomata function during *Puccinia graminis* penetration can effect penetration efficiencies thus penetration is not always solely dependent on light, demanding more thorough retrospection of factors influencing pathogen establishment^13^.

In barley, *Rpg1* effectively provided broad spectrum resistance to many races of stem rust in the upper Great plains of the United States and Western Canadian Prairies^14^ until broken by *Pgt* race QCCJB in 1989 and later the highly virulent *Pgt* race TTKSK, infamously known as Ug99. A thorough barley germplasm screen identified effective *Pgt* race QCCJB and TTKSK resistance in the unimproved barley line Q2186, conferred by the *rpg4*/*Rpg5* resistance locus designated as *rpg4*/*Rpg5*-mediated resistance locus (RMRL)^15^. RMRL harbors three tightly linked genes *(Rpg5*, *HvRga1* and *HvAdf3*) and provides resistance against a broad spectrum of stem rust races including *Pgt* race TTKSK^16,17^. Interestingly, a study of 73 landraces collected from the mountainous region of Switzerland screened with *Pgt* race TTKSK to identify new sources of resistance resulted in an unexpectedly high frequency of resistance (>43%) to *Pgt* race TTKSK and QCCJB^4^. The major contributor of *Pgt* race TTKSK resistance in the Swiss Landraces was RMRL. Thus, in the wake of the threat due to the spread of this virulent *Pgt* race and its lineage from Africa to Europe^18^ it is particular important to understand the host pathogen interactions that determine compatible and incompatible interactions with its cereal hosts wheat and barley^19^. These analyses require robust histological methodology to gain a thorough understanding of the spatial and temporal occurrences of these host-pathogen interactions that determine compatibility or incompatibility. However, due to the obligate biotrophic nature of *Pgt*, it has been impossible to transform with fluorescent tags for microscopic studies. Further, current methods for fungal staining are not robust as there is difficulty in getting adequate penetration of fluorescence dyes through the cuticle waxy layers on the leaf surface presenting a major hurdle to visualizing pathogen structures developing inside the plant tissues. To facilitate the penetration of dyes through the leaf epidermis, previously published protocols^20^ included autoclaving the leaf samples for 15 minutes at 121 °C in 1M KOH supplemented with a wetting agents such as Silwet-L77. However, this harsh treatment leads to very fragile leaf tissue after autoclaving that results in the disruption of a high proportion of samples during transfer to microscopic slides and the staining process. Thus, generates a high proportion of less than optimal samples resulting in wasted consumables, time and inconsistent images. Calcofluor white and Uvitex 2B are commonly used fluorescence dyes in clinical and plant studies, although Calcofluor white fades quickly and is not very efficient after counterstaining^21^ and the use of formalin as a specimen fixative in Uvitex 2B^22,23^ protocols drastically reduces fluorescent intensity^24^.

To visualize *Pgt* infection structures at the very early stages of infection and colonization we developed a refined method to process, fix and clear leaf samples. The developed method for autoclaving, washing, staining, and mounting on microscope slides, avoids possible sample disruption during handling prior to visualization under the microscope. The method also uses small amounts of costly staining solution. Using this method, we demonstrated that *Pgt* penetrates stomata guard cells of barley and wheat gaining forced entry during the initial dark period, 16–24 hours post inoculations (HPI). This is in sharp contrast to previous interpretations of the stem rust infection process, which was determined by visual phenotyping post inoculation with extended dark cycle treatments. Interestingly, the branching of PIH in the substomatal spaces and apoplast were profuse in the normal dark-light cycle treated plants whereas the dark treated plants showed inhibited growth with significantly less intercellular volume than the pathogen colonies growing under the normal light cycle. Thus, we present a robust new in planta fungal staining procedure that allowed for the elucidation of the penetration and colonization processes of *P*. *graminis* on the cereal hosts which until now was lost in the dark.

## Results

### Dark periods produce phenotypic variation among barley genotypes

**Figure 1:**
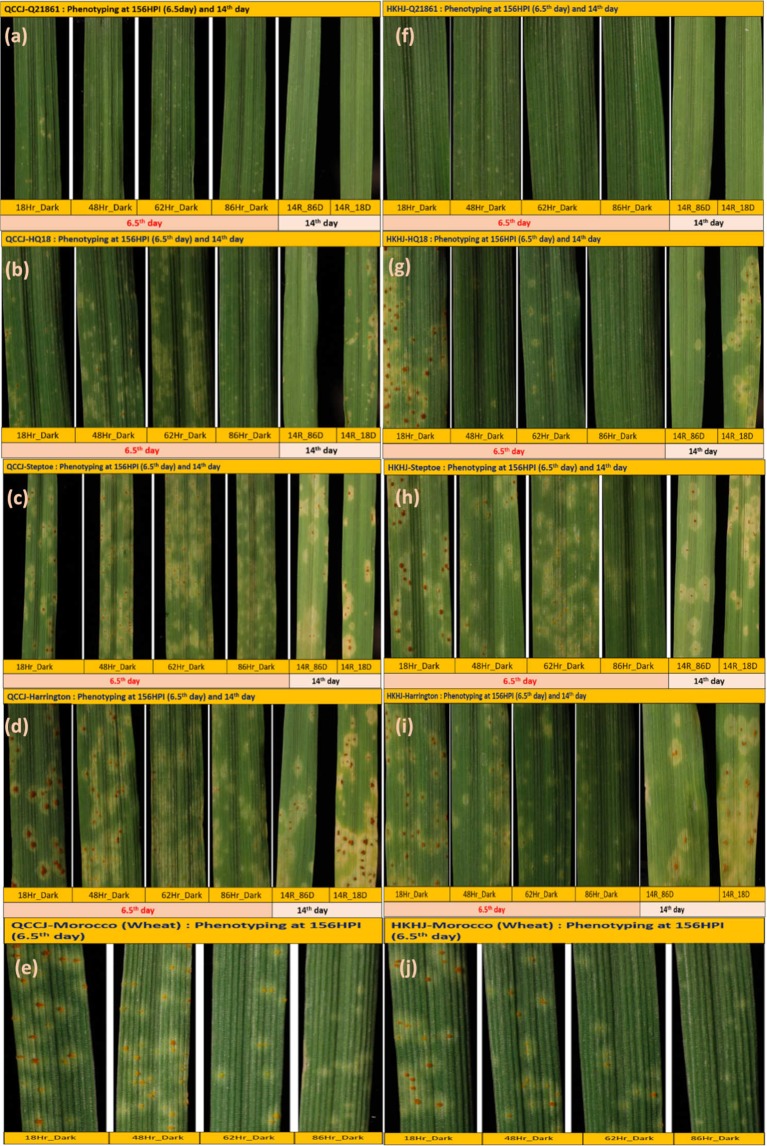
Disease phenotyping of differential barley genotypes. The barley lines Q21861 (RMRL+/*Rpg1*+), Steptoe (rmrl−/*rpg1*−), near isogenic line HQ18 (RMRL+/*rpg1*−), and Harrington (rmrl−/*rpg1*−*)* and the wheat line Morocco were inoculated with *P*. *graminis* f. sp. *tritici* (*Pgt*) race QCCJB (avirulent on RMRL but virulent on *Rpg1* containing barley genotypes) and HKHJC (avirulent race on *Rpg1* but virulent on RMRL containing barley genotypes). The four different sets of inoculated barley seedlings were given 18, 48, 62 and 86 hours of dark post inoculation with *Pgt* races QCCJB or HKHJC followed by resuming the normal light cycle of 16 hours light and 8 hours of dark in an environmentally controlled growth chamber. To assess difference in disease progression phenotyping was performed at 156 hours post pathogen inoculation and on the 14^th^ day post inoculation. (**a**–**e**) Disease phenotype with different light/dark treatments on the four barley genotypes and susceptible wheat line inoculated with *Pgt* race QCCJB. (**f**–**j**) Disease phenotype with different light/dark treatments on the four barley genotypes and susceptible wheat line inoculated with *Pgt* race HKHJC.

Phenotypic variation was assessed at 3, 6.5- and 14-days post inoculation (DPI) with *P*. *graminis* f. sp. *tritici* (*Pgt*) races QCCJB and HKHJC. After 3 DPI no visible leaf symptoms were observed on any of the tested genotypes, which included the barley, lines HQ18 (*rpg1*−/RMRL+), Q21861 (*Rpg1*+/RMRL+), Harrington (*rpg1*−/rmrl−), and Steptoe (*rpg1*−/rmrl−), and the wheat line Morocco (a *Pgt* universal susceptible wheat line). At 4.5 DPI onward, small pinpoint chlorotic spots were observed on the leaf surface of susceptible genotypes Steptoe, Harrington and Morocco for the *Rpg1* virulent *Pgt* race QCCJB; and Steptoe, Harrington, HQ18 and Morocco for the RMRL virulent *Pgt* race HKHJC, when provided the normal light cycle. Seedlings subjected to the extended dark periods (48, 62 or 86 hours post inoculation) showed no visible symptoms on their leaves (Fig. 1). From 6.5 DPI onwards, rust pustules became visible and started forming on the susceptible genotypes inoculated with either *Pgt* races QCCJB or HKHJC when exposed to the normal light cycle or the 48 hour (hr) extended dark period. However, plants treated with the 48-hr dark period had smaller pustules with more chlorosis and coalescing chlorotic halos surrounding smaller light orange-brown colored pustules when compared to the normal light cycle treated controls (Fig. 1). The 62-hr and 86-hr dark treated susceptible plants started showing large diffused chlorotic spots on the leaf surfaces without the formation of pustules at 6.5 DPI, indicating intercellular pathogen growth and colonization.

### Optimized confocal microscopy method to visualize and calculate bio-volume of fungal structures *in planta*

**Figure 2:**
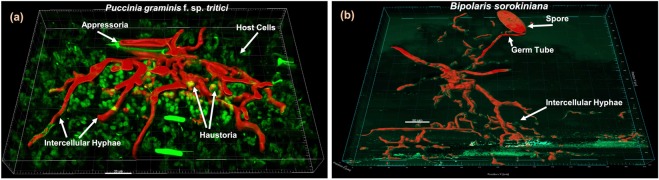
Surface creation and bio-volume analysis for (**a**) *Puccinia graminis* f. sp. *tritici* race QCCJB and (**b**) *B*. *Sorokiniana* isolate ND85F.

The method developed in this study for fixing and clearing leaf samples still relies on autoclaving, washing, staining, and mounting on microscope slides, but avoids sample disruption due to handling, providing consistent preparation of pristine samples prior to visualization under the microscope. The samples prepared using the method also had higher signal, thus, detection was far superior to the conventional staining and imaging techniques, enabling visualization, surface creation and bio-volume analysis using Imaris software (Imaris 9.0). We tested the efficiency of the new method with the biotrophic pathogen *Puccinia graminis* f. sp. *tritici* (Fig. 2a, Supplementary Fig. S1) and the necrotrophic pathogen *Bipolaris sorokiniana* (Fig. 2b) and obtained quality images for both pathogens despite their different lifestyles, biotrophic and necrotrophic, respectively.

### Confocal microscopy to visualize *Pgt* growth in planta during variable dark periods

**Figure 3:**
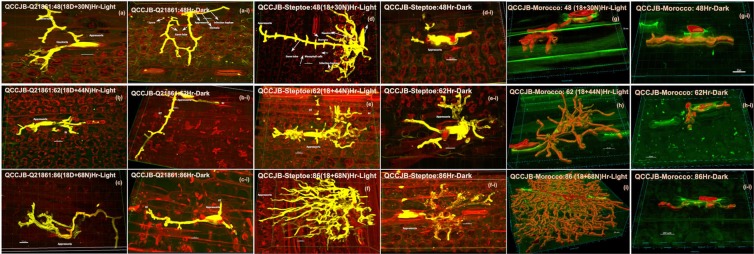
Microscopic visualization of *P*. *graminis* f. sp. *tritici* (*Pgt*) race QCCJB structures on cereal hosts treated with normal (18 hr initial dark period followed by 16/8 hr light/dark cycle) and continuous dark for 48, 62, and 86 hours post inoculation (HPI). (**a**–**c**: Normal growth cycle, **a**–**i** to **c**–**i**: continuous dark) Infection structures inside the resistant barley line Q21861 (RMRL+/*Rpg1*+). (**d**–**f**: Normal growth cycle, **d**–**i** to **f**–**i**: continuous dark) Infection structures inside the susceptible barley line Steptoe (rmrl−/*rpg1*−). (**g**–**i**: Normal growth cycle, **g**–**i** to **i-i**: continuous dark) Infection structures inside the susceptible wheat line Morocco. The *Pgt* structures in barley were visualized using Alexa Fluor-488-WGA dye shown in yellow, with the auto fluorescence of background plant structures shown in red. In wheat the pathogen is shown and volume constructed in red with the auto fluorescence of background plant structures shown in green. *Puccinia graminis* f. sp. *tritici* race QCCJB substomatal structures show that the pathogen was able to penetrate the cereal stomata irrespective of the presence or absence of light at 48 HPI. A reduction in branching and haustoria formation was observed when both resistant and susceptible seedlings were exposed to continuous dark periods post inoculation. Size bars represent 20 micrometers. GT - Germ Tube, A - Appressoria, IH - Infection Hyphae.

Confocal microscopy was used to determine if *Pgt* can effectively enter its cereal hosts through stomata without exposure to light. The resistant line Q21861, under the normal 18-hr dark period after pathogen inoculation followed by 16-hr light/8-hr dark cycle, had all the *Pgt* structures that normally form during the first phase of the biphasic infection cycle including the germinated spore, germ tube, and appressoria. All these structures were clearly visible on the leaf surface at all the time-points tested (Fig. 3a–c). The Q21861 48 hours post inoculation (HPI) samples showed that stomata penetration was followed by sub-stomatal vesicle and primary infection hyphae formation, and limited substomatal hyphal branching (Fig. 3a). However, in Q21861, which contains the *Rpg1* gene that provides effective resistance against *Pgt* race HKHJC and the RMRL providing resistance against *Pgt* race QCCJB, the overall branching and haustoria formation was contained to mesophyll cells adjacent to the breached stomata and did not significantly spread at the later time points (Fig. 3b,c).

**Figure 4:**
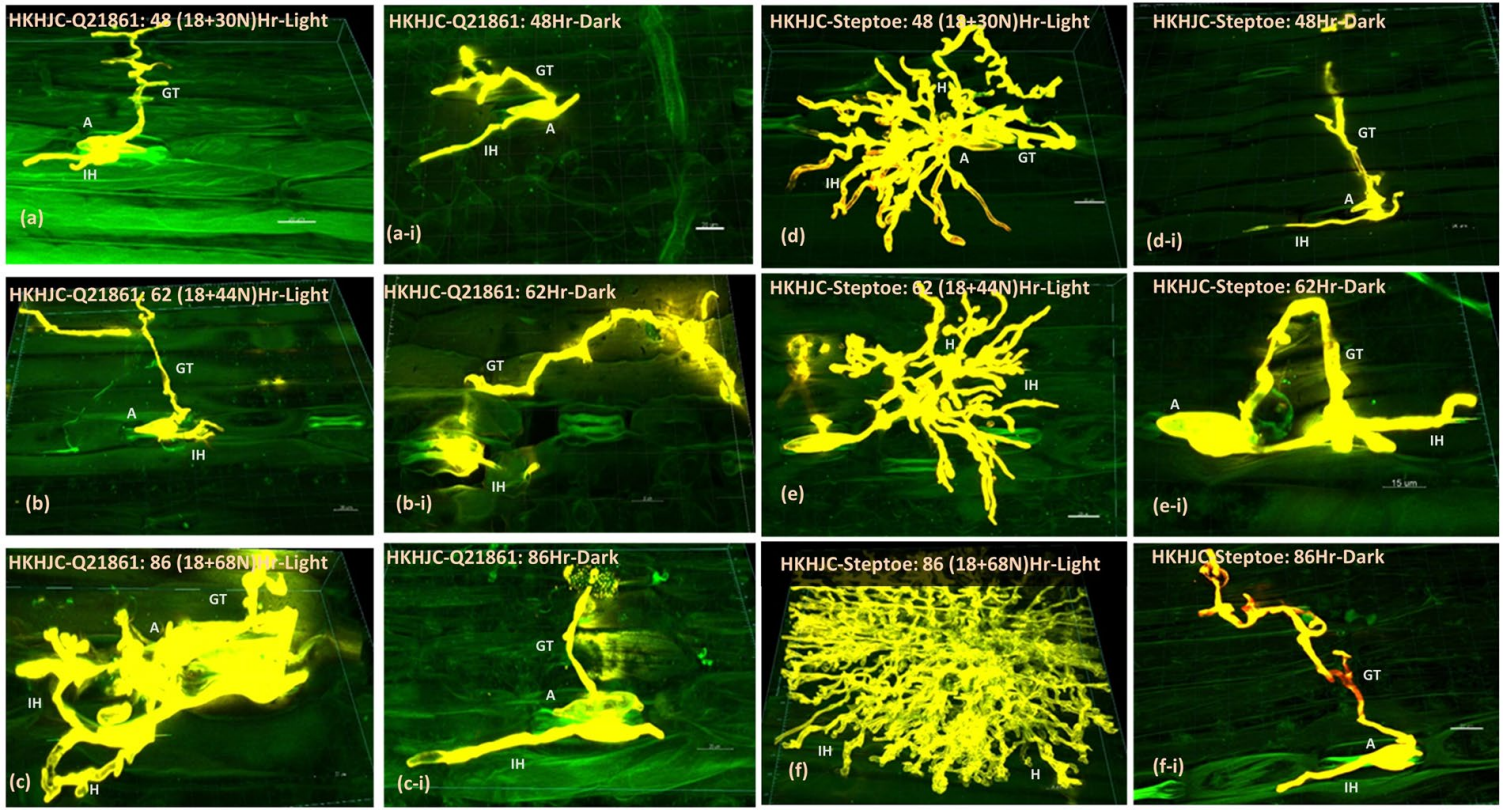
Microscopic visualization of *P*. *graminis* f. sp. *tritici* (*Pgt*) race HKHJC structures inside the resistant barley line Q21861 (RMRL+/*Rpg1*+) and susceptible barley line Steptoe (rmrl−/*rpg1*−) at 48, 62 and 86 hours post inoculation (HPI). Inoculated Q21861 and Steptoe plants were treated with (**a**–**f**) normal (18 hr initial dark period followed by 16/8 hr light/dark cycle) and (**a**–**i** to **f**–**i**) continuous dark periods (48, 62, 86 HPI). The *Pgt* structures in barley were visualized using Alexa Fluor-488-WGA dye shown in yellow, with the auto fluorescence of background plant structures shown in green. *Puccinia graminis* f. sp. *tritici* race HKHJC substomatal structures show that the pathogen was able to penetrate the cereal stomata irrespective of the presence or absence of light at 48 HPI. A reduction in branching and haustoria formation was observed when both resistant and susceptible seedlings were exposed to continuous dark periods post inoculation. Size bars represent 20 micrometers. GT - Germ Tube, A - Appressoria, IH - Infection Hyphae, H - Haustoria.

*Pgt* race QCCJB, produced profuse branching of intercellular hyphae (ICH) with extensive haustoria formation that spread deep into the mesophyll cell layers by 48 HPI in the susceptible barley line Steptoe when the 18-hr initial dark period was followed by the normal 16/8-hr light/dark cycle (Fig. 3d). At the later 62 and 86 HPI stages the growth of the pathogen was found to increase rapidly, indicative of a compatible interaction (Fig. 3e,f). In the wheat line Morocco, similar observations were made at 48 and 62 HPI (Fig. 3g,h, Movie S1), however, the branching of *Pgt* race QCCJB was more profound in wheat at the 86 HPI timepoint (Fig. 3i). In the resistant barley line Q21861 (Fig. 3a–i,b–i,c–i), susceptible barley line Steptoe (Fig. 3d–i,e–i,f–i), and susceptible wheat line Morocco (Fig. 3g–i,h–i,i-i), extended continuous dark periods post inoculation showed *Pgt* race QCCJB penetration of stomata, formation of substomatal vesicles and primary infection hyphae, that were present at all time points. These data indicated that penetration through the stomata was clearly light independent. However, hyphal branching and pathogen growth past the sub-stomatal vesicles formation stage was significantly reduced with minimal haustoria formation compared to the samples subjected to the normal light/dark cycle. This indicated an important role of light in the induction of pathogen growth, branching and haustoria formation after initial stomata penetration. Similar observations were made for *Pgt* race HKHJC inoculated Q21861 and Steptoe (Fig. 4) with the branching and haustorial formation of *Pgt* race HKHJC being severely impeded in the susceptible genotypes across all the time points subjected to continuous dark.

**Figure 5:**
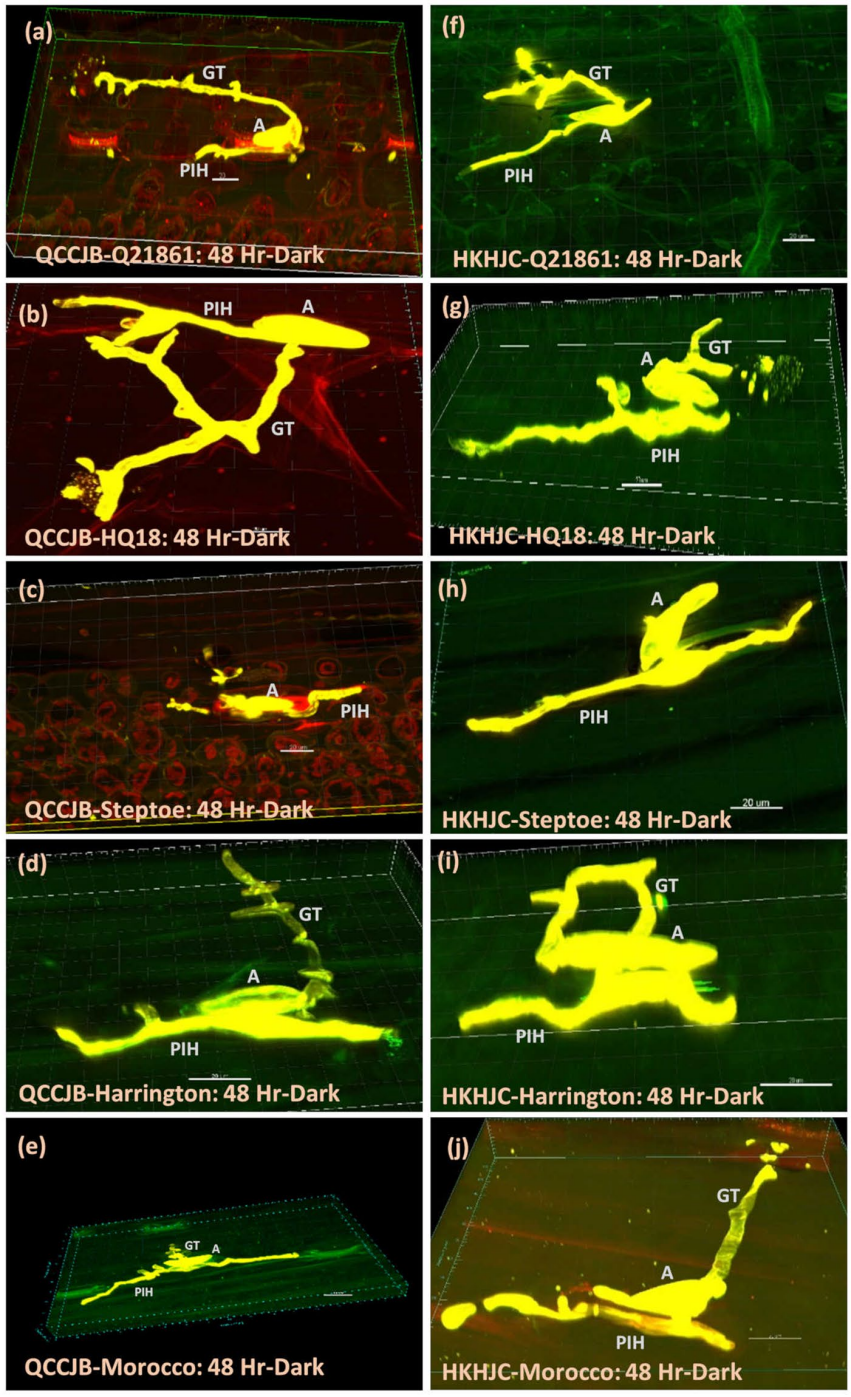
Light independent penetration of *Pgt* races QCCJB (**a**–**e**) and HKHJC (**f**–**j**) through host stomatal pore in barley line Q21861, HQ18, Steptoe, Harrington and the wheat line Morocco. Size bars represent 20 microns. GT - Germ Tube, A - Appressoria, PIH – Primary Infection Hyphae.

The near isogenic line, HQ18 (RMRL+/*rpg1*−), that is resistant to *Pgt* race QCCJB but susceptible to *Pgt* race HKHJC because of the introgression of RMRL into the susceptible line Harrington background and the susceptible line Harrington (rmrl−/*rpg1*−) also showed stomata penetration during extended dark treatment further validating the light independent penetration hypothesis. However, HQ18 and Harrington were only subjected to the 48-hr dark period post inoculation with *Pgt* races QCCJB or HKHJC (Fig. 5).

### Volume analysis

Fungal bio-volume was analyzed for three different infection sites representing the general growth patterns for *Pgt* races QCCJB and HKHJC in barley and wheat leaves at three different time points (48, 62 and 86 HPI) using the Imaris 9.0 surface creation function. Surface creation allowed for clear visualization of the pathogen’s intercellular structures and intracellular haustoria, and also facilitated the computation of the total bio-volume of pathogen present inside the host for each individual infection site. Volume analysis clearly showed an increase in pathogen growth over time when optimum growth conditions were provided. The normal light cycle treated barley line Steptoe and wheat line Morocco (18-hr initial dark period followed by 16/8-hr light/dark cycle) showed a rapid increase of pathogen bio-volume at 62 HPI to 86 HPI. This rapid increase of pathogen bio-volume was expected for the compatible interactions in susceptible hosts.

**Figure 6:**
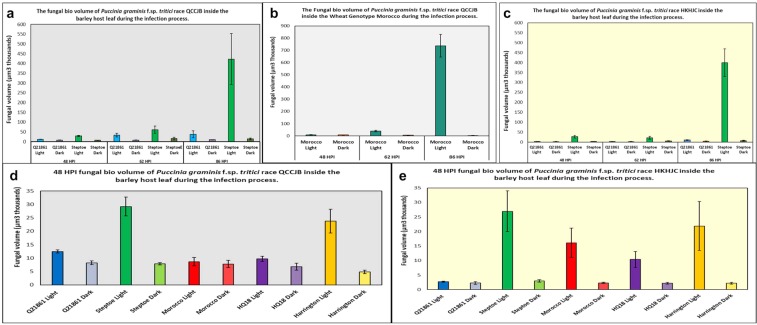
Bio volume calculation for fungal pathogen *P*. *graminis* f. sp. *tritici* (*Pgt*). Bio-volume calculation at the three time points (48, 62 and 86 hours post inoculation) under normal and extended dark period treatments of the resistant barley line Q21861 (RMRL+), susceptible barley line Steptoe (rmrl−) and susceptible wheat line Morocco inoculated with *Pgt* races QCCJB (**a**,**b**) and HKHJC (**c**). 48 HPI biovolume was calculated for Q21861, Steptoe, Harrington, HQ18 and Morocco for *Pgt* races (**d**) QQCJB and (**e**) HKHJC. The X-axis shows the times leaf tissue sample collections for the different barley lines and wheat line with the normal light/dark cycle (18-hour initial dark period followed by 16/8 hr light/dark cycle after pathogen inoculation) and extended continuous dark periods (48, 62, 86 hours) followed by 18 hours of initial moist dark period after pathogen inoculation. The y-axis represents average biovolume of *Pgt* race QCCJB computed for three infection sites in thousand-µm^3^. Error bar represents standard error of mean (N = 3).

Fungal bio-volume was calculated for three different infection sites exhibiting the typical growth patterns observed for fifteen infection sites (Table S1). The means and standard errors were calculated at each time point using the data for each of the three observed infection sites (Table S2, Fig. 6).

### Cost effectiveness of developed staining method

**Figure 7:**
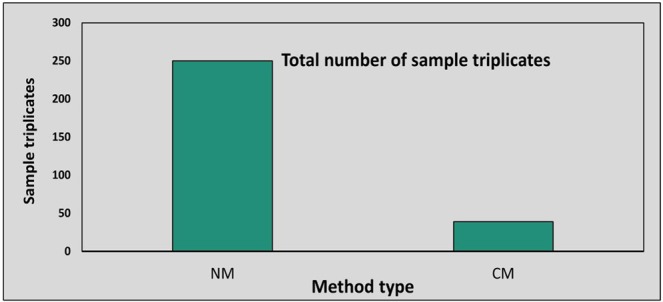
A comparison of new method (NM) with conventional methods (CM) for total number of sample slides using 20 µg/ml WGA-AF488. More than six times the number of samples can be processed using equal amount of stain.

The improved methodology developed not only increases the sensitivity of pathogen structure detection due to limited sample disruption but also significantly lowers the amount of expensive reagents consumed. The cost of WGA-AF488 is a major consideration when designing experiments, as it is a relatively expensive fluorescent stain. In conventional methods, at least 15–20 ml of staining solution (20 µg WGA-AF488/ml) is required for proper submerging of the leaf samples being stained in small petri plates or 50 ml polypropylene tubes. Thus, approximately thirteen samples can be processed when using three biological replicates, totaling 39 individual samples, with 5 gm of WGA-AF488 constituting 250 ml of staining solution (20 µg/ml). The new method uses only 350 µl of staining solution for each sample slide, thus; approximately 715 individual slides can be processed using 5 mg of WGA-AF488 (Fig. 7). Although processing multiple samples together with the conventional methods can reduce the volume of staining solution per sample the difference remains remarkable. Thus, the method described facilitates the generation of consistent high-quality images and accurate pathogen volume analysis at a much lower cost compared to the previously described methods. Further, the method for biovolume calculation can be used to understand and quantify the progression of pathogen colonization and growth inside the host.

## Discussion

Stomata penetration by the biotrophic fungal pathogen *Puccinia graminis* (*Pg*) was concluded to be light dependent in previous studies that reported negligible percentages of colony establishment by appressoria on susceptible wheat cultivars exposed to extended dark periods post inoculation^6,8^. However, this historic interpretation based on visible phenotyping data had been incorrectly maintained and needed to be revisited. Previously described WGA conjugated fluorescent staining methods^20,22,23^ for sensitive detection of fungal pathogen involve a boiling step disrupting the sample integrity thus reducing the sensitivity of an otherwise efficient method. The need of highly sensitive and less disruptive detection method for fungal pathogens, especially those that are recalcitrant to transformation, in order to characterize the early/limited spatial and temporal development of substomatal structures, made it necessary to develop an improved method preserving the sample integrity for *in-planta* fungal staining. 3D phenotyping of three fungal pathogens on maize was previously described using two photon microscopy^25^ and fungal volume assessment was reported for mycorrhizal fungi^26^. Here we report on the development of an improved, cost effective and rapid method of fungal WGA-AF based staining, visualization, and bio-volume analysis of *in-planta* pathogens that works irrespective of their life style, biotroph or necrotroph, or their recalcitrance to transformation. We then utilized the method to show that stomata penetration by *Pgt* to its cereal host is light independent.

The effects on the initial establishment of *Pgt* races QCCJB and HKHJC exposed to extended initial dark periods post inoculation was investigated on two cereal hosts, barley and wheat. Phenotypic observations of the susceptible lines, Steptoe, Harrington and Morocco, subjected to the normal light/dark cycle post *Pgt* inoculation showed development of chlorotic specks on the primary leaves by the 4th to 5th DPI (100–110 HPI), with small sporulating pustules developing by the 5.5 to 6th DPI (130–140 HPI) and susceptible pustules with spores erupting through the epidermis by 11th to 14th DPI. These observations determined that visible phenotypes developed on leaf surface approximately 118–130 hours post exposure to light after the initial 18-hr dark period following inoculation. Upon inoculation with *Pgt* races QCCJB or HKHJC with extended dark periods of 30 or 36 HPI, the patterns of observed phenotypes were similar to those of disease progression for normal light/dark cycle treated plants. These phenotypic observations indicated that the initial phase of intercellular pathogen growth is slow, and the effects of extended dark periods are not initially pronounced. However, extended dark periods of 48, 62, and 86 HPI slowed the visual phenotypes in comparison to the normal light cycle treated plants. Also, once the infection types appeared on the extended dark treated seedlings, the symptoms were phenotypically different than lesion development on the normal light/dark treated plants, having larger more diffuse chlorotic regions. The susceptible genotypes given 62 hours of continuous dark showed large chlorotic lesions at 115–120 HPI and started sporulation at 140–145 HPI (Fig. 1), which was only ~10 hours behind the normal light /dark treated plants. The susceptible plants given 86 hr continuous dark developed large coalesced chlorotic lesions, after 110–120 HPI and developed small light brown sporulating pustules at 145–150 HPI. Interestingly, visible sporulation appeared at 83–88 hours on susceptible genotypes given the 62 hour extended dark period and 59–64 hours on the 86-hr dark treated seedlings after exposure to the normal light period (16/8-hr light/dark period). On the normal dark/light treated seedlings sporulation occurring at 112–122 HPI. These data indicated that *Pgt* penetration is probably light independent, and that some initial substomatal growth occurs in the absence of light. Thus, we hypothesize that *Pgt* penetrates through the stomatal barrier during the dark period although the intercellular pathogen growth should be severely impeded resulting in delayed visible spore production.

To test the hypothesis that the pathogen breaches the stomata during the dark required visualization of substomatal structures during extended dark treatment post inoculation. However, initial attempts to generate these data were suboptimal due to the difficulty of obtaining intact samples after slide preparation and inefficient fluorescent staining of the intercellular pathogen structures. Thus, out of necessity we developed a fungal staining and bio-volume calculation method using confocal microscopy that significantly enhanced the current methodology for staining, visualization and bio-volume analysis of fungal pathogens *in-planta*. The newly developed methodology preserves the sample integrity and significantly lowers the amount of expensive reagents. Our improved method facilitates the generation of consistent high-quality images and accurate pathogen volume analysis at a much lower cost compared to the previously described methods. Further, the method for biovolume calculation can be used to understand and quantify the progression of pathogen colonization and growth inside the host.

To further investigate the hypothesis that pathogens require the initial light cycle to enter through the stomata following the first phase of the infection process, we utilized the developed methodology to visualize the effects of extended initial periods of darkness after *Pgt* inoculation using the two *Pgt* races, QCCJB and HKHJC, inoculated on resistant and susceptible barley genotypes and a susceptible wheat line. According to previous literatures^1,5,6,8,27^, *Pgt* appressoria remain quiescent in the dark after forming on top of stomata, until the light induces stomatal opening facilitating penetration. If this hypothesis was correct, then extended dark period should not show *Pgt* substomatal growth post appressoria formation.

Microscopic observations of barley lines Q21861, HQ18, Steptoe, and Harrington and the wheat line Morocco inoculated with *Pgt* race QCCJB or HKHJC at 48 HPI in continuous dark showed *Pgt* penetration through host stomata and the formation of substomatal vesicles as well as limited ICH growth during the extended dark periods. The stomatal penetration occurred irrespective of host-pathogen genetic interactions that determine compatible or incompatible interactions (Fig. 5). *Pgt* race QCCJB inoculations on the resistant barley line Q21861 showed effective penetration through stomata but the overall ICH branching and formation of haustoria was suppressed compared to the susceptible barley line Steptoe at each time point (48, 62, 86 HPI) under the typical light/dark treatment. At 62 and 86 hours post pathogen inoculation, the ICH branching and haustoria formation in Steptoe increased rapidly, whereas in Q21861 pathogen growth was contained and limited to adjacent mesophyll cells near to the breached stomata without any substantial branching (Figs 3 and 4). Thus, resistance mechanisms in barley line Q21861 provided by the RMRL^4,16,28^ for *Pgt* race QCCJB and *Rpg1* for *Pgt* race HKHJC, effectively takes place post *Pgt* penetration of the stomata aperture and suppress pathogen growth both pre and post haustoria formation. These results are consistent with the barley dual kinase *Rpg1*-mediated resistance mechanism^29^. *Rpg1* has been shown to be phosphorylated within five minutes of inoculation with the avirulent *Pgt* race MCCF^30^ and this phosphorylation and subsequent degradation at 24 HPI^31^ are indispensable for the resistance response, indicating that an early pre-haustorial pathogen induced protein modification of Rpg1 may induce prehaustorial defense mechanisms. For the two different *Pgt* races (QCCJB and HKHJC) visual differences or a significant difference in bio-volume in the resistant barley Q21861 was not observed. However, in the susceptible barley line Steptoe the average biovolume at 86 HPI with normal growth conditions for HKHJC (399739 µm^3^) was found to be less than QCCJB (422619 µm^3^), yet not statistically significant due to large variation in the intercellular growth of replicates. Interestingly, microscopy data showed deeper penetration and branching of QCCJB in Steptoe comparing to HKHJC at 86 HPI (See Supplementary Fig. S2), thus producing more bio-volume and suggesting that *Pgt* race QCCJB, the surrogate North American race for the virulent *Pgt* race TTKSK (a.k.a. Ug99), may be more aggressive than HKHJC. In the wheat line Morocco, the average bio-volume for *Pgt* race QCCJB (737514 µm^3^) was significantly higher than that of the susceptible barley line Steptoe (422619 µm^3^) indicating that this *Pgt* race maybe better adapted to wheat. In general, *Pgt* is more adapted to wheat but analysis of diverse *Pgt* isolates could validate this hypothesis based on the bio-volume methodology.

Plants with continuous 48-hr dark treatment, showed that *Pgt* race QCCJB intercellular growth in the susceptible barley line Steptoe was contained to 3–4 laterally adjacent cells in relation to the stomata, whereas extensive intercellular pathogen growth was observed in the normal light/dark treated plants. The visual differences of *Pgt* race QCCJB branching and haustoria formation were more pronounced when seedlings were subjected to extended dark periods of 62 and 86 HPI (Fig. 3b–i,c–i,e–i,f–i,h–i,i-i) compared with the pathogen growth on the normal light cycle treated plants (Fig. 3b,c,e,f,h,i). The continuous dark treated seedlings post inoculations had intercellular pathogen growth primarily contained to the upper mesophyll cell layers with little ICH branching and few haustoria. Similar results were obtained for *Pgt* race HKHJC (Fig. 4) on all tested genotypes, confirming that stomata penetration is light independent, yet pathogen growth including ICH branching and haustoria formation is induced by the light even in susceptible genotypes. Microscopic visualization conclusively shows that the stem rust pathogen can penetrate barley and wheat stomata in the dark, thus does not require light induced stomatal opening. However, light is required for *Pgt* colony development. These results indicate that *Pgt* penetration is similar to leaf rust pathogen *Puccinia recondita* which also does not require light periods for stomatal penetration^9^. Similarly, stomatal deregulation was reported in grapevine leaves infected with *Plasmopara viticola* irrespective of continuous darkness^32,33^. However, fungal pathogens such as *Cercosopra zeae-maydis* requires 12 hour light/dark cycle for successful infection of its host entering through leaf stomata. In constant darkness, *Cercosopra zeae-maydis* is unable to form appressoria on the stomata which is needed for subsequent stomatal tropism, indicating light is an absolute requirement for pathogen establishment and disease^34^. Thus, gaining entry through stomata and its light dependency is dependent on the pathogen’s physiology and differs for diverse pathogens. In our experiments we have found that *Pgt* race HKHJC showed a severely impeded branching and growth in the dark on the susceptible line Steptoe comparing to *Pgt* race QCCJB indicating that QCCJB may be more aggressive on Steptoe, further compounding the effect of darkness depending on the *Pgt* races.

Biovolume analysis was also utilized to determine differences among the resistant or susceptible barley lines for *Pgt* colony development. In the normal light/dark treated seedling leaves the overall volume of pathogen growth between line Q21861 and Steptoe was significantly different at 48 HPI (P < 0.05, N = 3). However, at 62 HPI and 86 HPI the difference in pathogen volume was not significant (P < 0.05, N = 3) despite large visual difference in the intercellular growth. We found large variability in the *Pgt* growth volume for the three analyzed (Tables S1 and S2) and many visualized infection sites. Typically, the number of successful colonies that form sporulating pustules represent a small percentage of the successful *Pgt* penetration sites on the leaf of susceptible host genotypes. Previously it was shown that suceptible barley cultivar Steptoe exhibits early resistance response for *Pg* race MCCF up to 48 HPI comparing with the resistant cultivar Morex, yet allows the rapid growth of pathogen at later time points^35^. Thus, it is possible that *Pgt* subcellular progression is suppressed at many infection sites even in the susceptible genotypes possibly representing a basal resistance mechanism that the pathogen effectively outgrows at some infection sites in susceptible genotypes lacking race specific resistance mechanisms. It is not known and was not determined here if this can be attributed to the timing of spore germination or the distance the germ tube grew before it encountered a stomata.

As expected, a small insignificant change in the pathogen growth and bio-volume was observed across all the infection sites analyzed between 62 HPI (33590 µm^3^) and 86 HPI (37975 µm^3^) in the barley line Q21861 inoculated with *Pgt* race QCCJB indicating growth suppression imparted by RMRL. For HKHJC similar pathogen growth arrest was observed in Q21861 indicating growth suppression imparted by *Rpg1*. However, in the susceptible line Steptoe, a 7.9-fold change in *Pgt* race QCCJB growth was noted between 62 HPI (61412 µm^3^) and 86 HPI (422619 µm^3^), confirming that this is a rapid stage of pathogen growth (Table S1) for majority of infection sites, yet some infection sites did not show this rapid growth as discussed earlier. For Steptoe, overall recorded *Pgt* race QCCJB biovolume for the normal light period was greater than Q21861, as expected due to its compatible interaction (susceptibility). Similarly, an 18-fold increase was detected by bio-volume analysis for *Pgt* race HKHJC in Steptoe from 62 to 86 HPI. We conclude that due to multiple branching of intercellular hyphae and rapid haustoria formation the pathogen is able to support its further growth in the susceptible cultivars. However, each successful infection does not necessarily have similar growth patterns, which leads to what is known as mesothetic reactions typically observed in barley.

Collectively, using an improved fungal pathogen visualization and adding a bio-volume calculation approach, we were able to show that *Pgt* races QCCJB and HKHJC can penetrate through stomata in the dark, although ICH branching and haustoria formation are drastically suppressed by prolonged initial dark treatment. However, exposure to light induces pathogen growth, suggesting that light is required to trigger the pathogen into the second colonization phase of ICH branching, growth and haustoria formation. Further, fungal bio-volume analysis across time points revealed that a rapid growth of *Pgt* takes place between 62 and 82 hours after pathogen spores land on the leaf surface of susceptible genotypes. Future studies will characterize if the reduction in pathogen growth due to the extended dark period is affecting plant biological processes that suppress pathogen growth or light is required to trigger changes in pathogen physiology and the next stages of colony development.

## Methods

### Plant material, pathogen inoculations procedure and growth chamber maintenance

**Figure 8:**
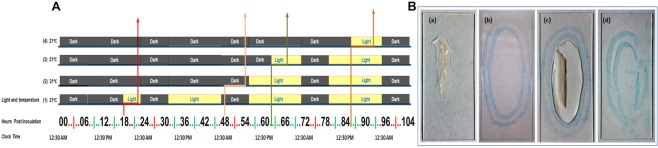
Growth conditions and sample preparation. (**A**) Growth chamber light conditions at different leaf sample collection time after *P*. *graminis* f. sp. *tritici* races QCCJB and HKHJC inoculations on host plants. Arrows indicate sample collection times i.e. 18 HPI, 48 HPI, 62 HPI and 86 HPI at different light conditions represented by horizontal bars applied to seedlings used for phenotyping at ~6.5 and 14 days post inoculation. The later three-time points 48, 62 and 86 HPI were used for sample collection for fluorescence microscopy. (**B**) Barley leaf tissue sample processing on glass slides. (a) Fragile barley leaf section transferred to glass slide after autoclaving in 1M KOH. (b) Hydrophobic barrier made by ImmeEdge PAP pen on the glass slide. (c) Hydrophobic barrier containing a barley leaf sample, buffer and staining solution inside the ImmeEdge PAP pen hydrophobic barrier (d) Disrupted hydrophobic boundary due to autoclaving and KOH.

Five plant genotypes including two *P*. *graminis* f. sp. *tritici* susceptible and two resistant barley lines and the susceptible wheat line Morocco, were used in the study. The universal susceptible barley lines Steptoe (rmrl−/*rpg*−) and Harrington (rmrl−/*rpg*−), and the highly resistant line Q21861 (RMRL+/*Rpg1*+) were used in the study. The resistant barley line Q21861 is resistant to all known stem rust pathotypes. A resistant Near Isogenic Line (NIL) HQ18 (RMRL+/*rpg1*−) was created by transferring the *RMRL* into the Harrington genetic background (Harrington x Q21861) by repetitive backcrossing to the BC_6_ generation. Two different *P*. *graminis* f. sp. *tritici* races, QCCJB (virulent on *Rpg1*) and HKHJC (virulent on RMRL), were used^4,36^. The RMRL provides effective resistance against *Pgt* race QCCJB^28^, and *Rpg1* provide resistance to *Pgt* race HKHJC^29^. Growth chambers for plant maintenance were set to provide 16 hours of light starting from 7 A.M. to 11 P.M. and 8 hours of dark from 11 P.M. to 7 A.M at 21 °C (Fig. 8). Plants were inoculated at 12:30 A.M., after shutting down of growth chamber lights providing 1.5 hours of dark period before pathogen inoculation to ensure closing of leaf stomata.

Nine-day old barley and wheat plants were inoculated with stem rust urediniospores on fully expanded primary leaves. 10 mg/ml of urediniospores mixed in lightweight mineral oil (Soltrol 170 isoparaffinic oil, Chevron Phillips chemical company) was used to inoculate each 96-cone rack containing one seedling per cone. Approximately 800 µl of spore mixture was sprayed in the form of a fine mist for uniform inoculations. Plants were kept in a dark chamber at 100% humidity immediately after drying surface oil at 20 °C. After the initial incubation period of 18 hours dark at 100% humidity required for spore germination, one rack of inoculated seedlings was transferred into growth chambers providing normal growth conditions of 16 hours of light starting from 7 A.M. to 11 P.M. and 8 hours of dark from 11 P.M. to 7 A.M. Another rack of inoculated barley seedlings were given extended dark period treatment. We assayed seedlings exposed to continuous dark periods of 48, 62 and 86 hours post inoculation (HPI) before moving them to a growth chamber with the normal light cycle and growth conditions. Through the experiment temperature was maintained at ~20–21 °C.

### Collection, fixation and clearing of leaf samples

2.5-centimeter long cuttings from the middle portion of primary leaves were collected at each time point and transferred into anhydrous farmers fixative (Ethanol 3 parts: Glacial acidic acid 1 part) for fixing and leaf clearing. Samples were stored in fresh farmer’s fixative until processed for microscopy staining.

### Heat treatment of cleared samples for efficient stain penetration

Samples were removed from farmer’s fixative (FF) and washed in 30 ml 1x phosphate buffer saline (PBS) pH 7.5 supplemented with 0.05% Tween-20, twice for five minutes at 60 rpm in 50 ml polypropylene tubes. Sample were then washed two times for five minutes at 60 rpm with Tris HCl (pH 7.4) supplemented with 0.05% Tween-20 to remove residual FF from the tissues, which is critical before the staining process. Two concentric appropriate sized hydrophobic oval boundaries were drawn on the Histobond microscope glass slide (Fisher Scientific) using ImmeEdge hydrophobic barrier PAP pen (Vector Laboratories) (Fig. 8B–b) and a leaf sample was placed inside the boundaries keeping the adaxial surface of leaf sample facing up. The slide was transferred into an autoclavable rack for easy handling. 350 µl 1M KOH + 0.05% Tween-20 solution was carefully pipetted onto the leaf sample keeping the sample and solution inside the hydrophobic barrier (Fig. 8B–c). The hydrophobic barrier maintains the liquid solution on the glass slide covering the leaf sample keeping it hydrated and submerged. The rack with the slides on top was kept on a plate shaker at 50 rpm for three minutes, then the solution was replaced with fresh 1 M KOH + 0.05% Tween-20 solution using a 1 ml pipette and all the samples on rack were autoclaved using the liquid cycle for 15 minutes, at 121 °C and 15 PSI covered with aluminum foil. Covering prevents drying of slides and does not allow excess moisture to build up during the liquid autoclave cycle. After autoclaving the remaining KOH buffer was pipetted off the slides were cleaned with a Kim wipe to remove the distorted hydrophobic boundary (Fig. 8B-d) without disturbing the autoclaved leaf sample. Then a new hydrophobic boundary was applied encircling the sample. This slide autoclaving procedure avoids sample disruption mainly due to fragility because of sample boiling in excess KOH solution and subsequent sample transfer and handling (Fig. 8B–a). After applying the new hydrophobic barrier, the samples were washed twice with 1x PBS + 0.05% Tween-20 solution for 5 minutes at 50 rpm and the buffer solution was pipetted off.

### Sample staining and mounting

Samples were stained with a final concentration of WGA-Alexa Fluor 488 dye (20 µg/ml) dissolved in 1x PBS buffer supplemented with 0.05% Tween-20. For immediate staining 350 µl of dye was pipetted onto the samples inside the hydrophobic boundary after removal of the PBS buffer. This procedure allows for the use of a small amount of staining solution (350 µl) for each sample. Samples were stained for one hour at 50 rpm rotation at room temperature. However, 10–15 minutes of staining worked equally as well. Longer staining must be avoided as evaporation of buffer causes staining molecules to remain tightly bound to the leaf surface resulting in high background fluorescence. After staining, the samples were washed three times with Tris HCl pH 7.4 (without Tween-20) for 5 minutes at 60 rpm to remove the excess dye on the leaf surface. To prepare slides for confocal microscopy, the hydrophobic boundary was removed using a Kim wipe and Ecomount mounting media (Biocare Medical) was pipetted onto the sample, enough to cover the sample thoroughly, and a coverslip was carefully placed on the samples avoiding trapping of air bubbles. The slides were dried at room temperature overnight in the dark and stored in 4 °C until visualization under the microscope. We have observed that properly stored slides retain fluorescence for more than one year without losing fluorescence emittance efficiency.

### Image capturing and analysis

The prepared slides were visualized with a LSM 700 laser scanning confocal microscope using Plan-Apochromat 40x/1.3 oil immersion lens and 10x objective lens using 2 different channels (Zeiss Thornwood, NY). Green channel 488/520 was assigned for WGA-Alexa Fluor staining and red channel 555/580 was used for auto florescence detection. For inner structure visualization Z-stack images were taken depending upon the depth of stem rust structures developed inside the plant tissues (100–300 images per infection site up to 120 µm deep). Computation from Z stack was performed in ZEN (Zeiss Thornwood, NY) software to get a collapsed picture, and Imaris (9.0.1) (Bitplane, South Windsor, CT) software to obtain 3D reconstruction for volume analysis.

Images were finally processed in Imaris 9.0.1 software. Imported pictures were subjected to channel correction for red and green channel. Image processing was performed using attenuation correction for the green channel with intensity front 256 and intensity back 128 values. Smoothing for channel green was performed using median filter size 5 × 5 × 5. Processed images were subjected to contrast change using normalized layers function to remove the excess background. This process enabled the visualization of pathogen volume growth inside the plant cells while removing the background plant noise as recorded in the red channel. Once pathogen growth volume area was determined, using the surface creation function, the surface area of pathogen growth was created specifically for the green channel using surface detail value 0.491 at absolute thresholding. All nonspecific signals were removed using volume filtering. All the nonspecific surface creation was removed manually, and statistical analysis function on Imaris software was run for surface volume assessment using the total volume of fungal structures present from appressoria to in planta growth. It is important to create the surface avoiding overexposure, which causes inclusion of plant structures facilitating the loss of poorly stained pathogen structures and underestimation of the computed volume of pathogen structures. Thus, it is important to calculate volume as a function of average value for all the constructed surfaces.

### Volume analysis

Average fungal bio-volume for *Pgt* race QCCJB and HKHJC was recorded after surface creation in barley and wheat leaf samples collected at three different time points 48, 62 and 86 HPI with two different treatments, continuous dark and normal dark/light period, on Imaris 9.0.1 software. Average pathogen area was calculated for three different infection sites exhibiting a general growth pattern observed across fifteen infection sites. Means and standard-errors were calculated at each time points for three observed infection sites in Microsoft Excel. t-test was used to analyze statistical differences.

### Stem rust disease phenotyping

Phenotyping for pathogen signs and plant symptoms was performed at 6.5 DPI for 18 HPI, 48 HPI, 62 HPI and 86 HPI dark treated plants for presence of spore pustules and chlorotic spots on the leaves. Later at 14 DPI disease phenotyping was carried out using modified Stakman infection type (IT) scale used for barley^15,37^.

## Supplementary information

Supplementary File

Supplementary Movie S1
