# Supplementary material for: Shedding Light on Penetration of Cereal Host Stomata by Wheat Stem Rust Using Improved Methodology
Source: Sci Rep. 2019 May 28;9:7939. doi: 10.1038/s41598-019-44280-6 (PMC6538696; doi:10.1038/s41598-019-44280-6)
Supplement: Supplementary file 1 — Supplementary File [file 41598_2019_44280_MOESM1_ESM.docx]

**Shedding Light on Penetration of Cereal Host Stomata by Wheat Stem Rust Using Improved Methodology**

**Shyam Solanki^1^, Gazala Ameen^1^, Pawel Borowicz^2^, and Robert S. Brueggeman^1*^**

^1^Department of Plant Pathology, North Dakota State University, Fargo, ND 58108-6050, USA

^2^Department of Animal Sciences, North Dakota State University, Fargo, ND 58108-6050, USA

*Corresponding author: robert.brueggeman@ndsu.edu


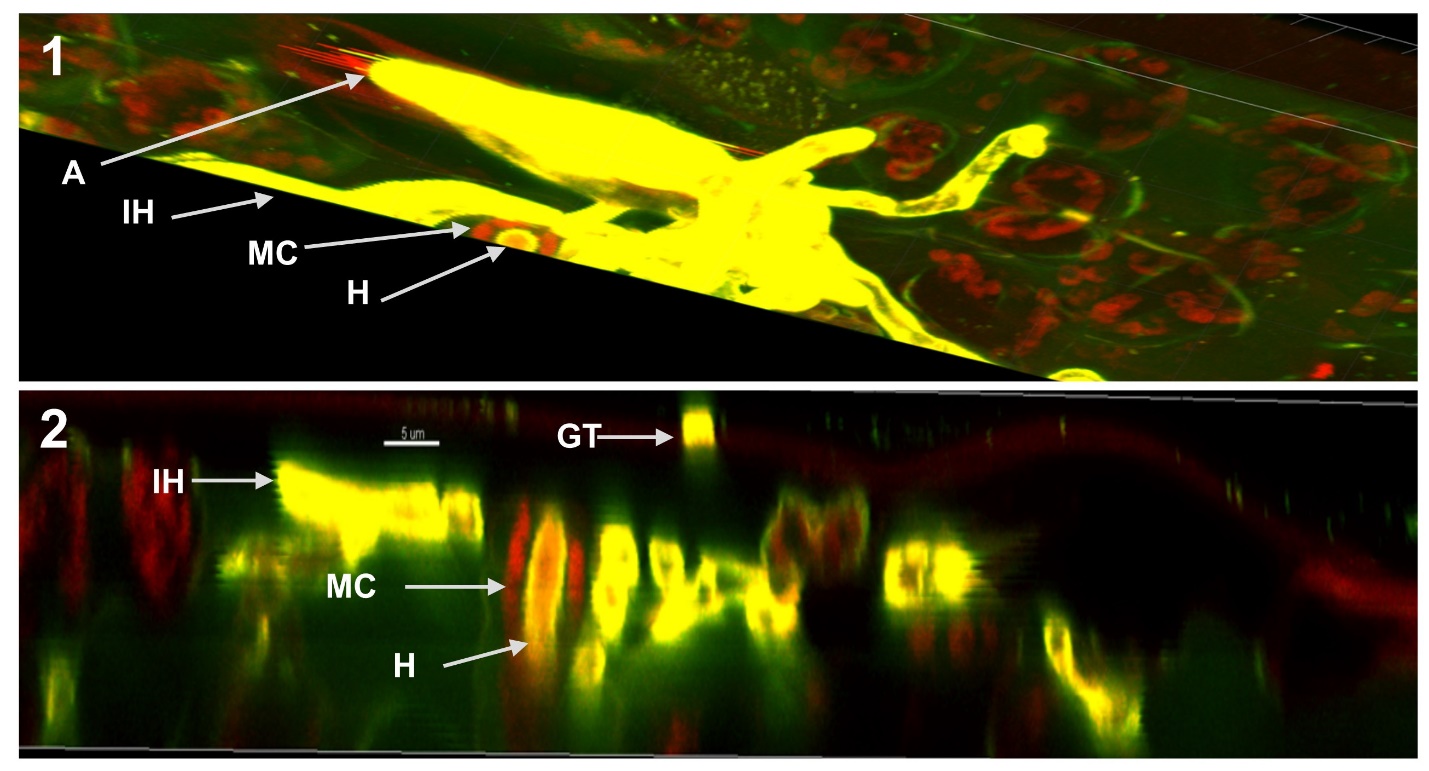


**Supplementary Figure 1.** Various infection structures of fungal pathogen *Puccinia graminis* f.sp. *tritici* on the barley leaf at 48 hours post inoculation shown in two different planes. GT – Germ Tube, A – Appressoria, IH - Infection Hyphae, H – Haustoria, MC – Mesophyll Cells (Barley).


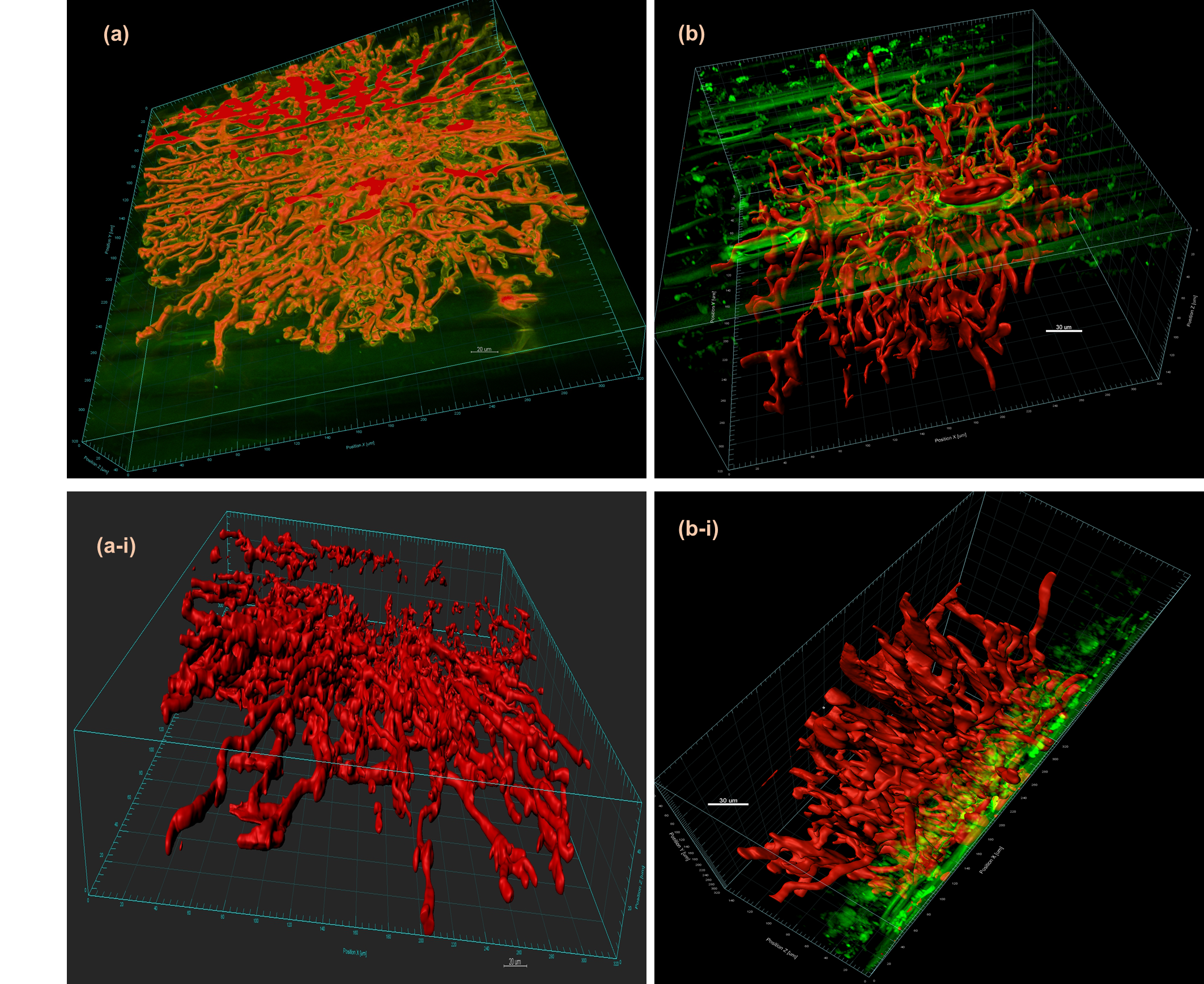


**Supplementary Figure 2.** Difference in penetration depth for HKHJC (**a, a-i**) and QCCJC (**b, b-i**) on susceptible barley Steptoe at 86 hours post inoculation with normal dark/light cycle after surface creation for a representative spore penetration site. Size bars represents 20 micrometers for HKHJC and 30 micrometers for QCCJC. Box represents the growth in XYZ direction in leaf in micrometers.

**Table S1.** The fungal surface bio volume (µm^3^) of *Puccinia graminis* f.sp. *tritici* race QCCJB inside the barley and wheat cereal hosts leaf during the infection process. Biovolume of infection sites are used to calculate mean and standard error in MS-Excel.

| Time points | Q21861 | mean | Std Error | Steptoe | Mean | Std Error | Morocco | Mean | Std Error |
| --- | --- | --- | --- | --- | --- | --- | --- | --- | --- |
| 48L | 12343 | 12445 | 618 | 37206 | 29198 | 3488 | 10546 | 8666 | 1533 |
| 48L | 13805 |  |  | 27774 |  |  | 10541 |  |  |
| 48L | 11188 |  |  | 22614 |  |  | 4910 |  |  |
| 62L | 22556 | 33590 | 10152 | 26880 | 61412 | 18524 | 29973 | 40543 | 6175 |
| 62L | 19809 |  |  | 53189 |  |  | 55198 |  |  |
| 62L | 58406 |  |  | 104168 |  |  | 36460 |  |  |
| 86L | 19766 | 37975 | 17396 | 490278 | 422619 | 129976 | 833057 | 737514 | 93121 |
| 86L | 80444 |  |  | 658212 |  |  | 869118 |  |  |
| 86L | 13717 |  |  | 119368 |  |  | 510366 |  |  |
| 48D | 9870 | 8234 | 702 | 6784 | 7824 | 440 | 6894 | 7777 | 1356 |
| 48D | 7870 |  |  | 8101 |  |  | 10992 |  |  |
| 48D | 6961 |  |  | 8587 |  |  | 5444 |  |  |
| 62D | 9147 | 7913 | 822 | 32906 | 16409 | 6752 | 6112 | 6112 | 636 |
| 62D | 8674 |  |  | 9169 |  |  | 4762 |  |  |
| 62D | 5918 |  |  | 7153 |  |  | 7462 |  |  |
| 86D | 11424 | 10032 | 1062 | 8101 | 14307 | 4818 | 4312 | 3051 | 516 |
| 86D | 11239 |  |  | 26104 |  |  | 2507 |  |  |
| 86D | 7432 |  |  | 8715 |  |  | 2334 |  |  |

**Table S2.** The fungal surface bio volume (µm^3^) of *Puccinia graminis* f.sp. *tritici* race HKHJC inside the leaf of barley and wheat cereal hosts during the infection process. Biovolume of infection sites are used to calculate mean and standard error in MS-Excel.

| Time points | Q21861 | mean | Std Error | Steptoe | Mean | Std Error | Morocco | Mean | Std Error |
| --- | --- | --- | --- | --- | --- | --- | --- | --- | --- |
| 48L | 2600 | 2708 | 229 | 32184 | 26975 | 7020 | 20038 | 16143 | 5006 |
| 48L | 3238 |  |  | 10179 |  |  | 24265 |  |  |
| 48L | 2285 |  |  | 38561 |  |  | 4127 |  |  |
| 62L | 3349 | 2793 | 457 | 10488 | 21915 | 7315 |  |  |  |
| 62L | 1675 |  |  | 15675 |  |  |  |  |  |
| 62L | 3356 |  |  | 39582 |  |  |  |  |  |
| 86L | 7482 | 10058 | 1578 | 566347 | 399739 | 69496 |  |  |  |
| 86L | 13840 |  |  | 286188 |  |  |  |  |  |
| 86L | 8851 |  |  | 346681 |  |  |  |  |  |
| 48D | 3500 | 2260 | 515 | 3966 | 2983 | 452 | 1967 | 2339 | 215 |
| 48D | 1843 |  |  | 2933 |  |  | 2711 |  |  |
| 48D | 1437 |  |  | 2051 |  |  |  |  |  |
| 62D | 3086 | 2154 | 664 | 4637 | 5584 | 1382 |  |  |  |
| 62D | 2841 |  |  | 8871 |  |  |  |  |  |
| 62D | 533 |  |  | 3243 |  |  |  |  |  |
| 86D | 3259 | 4188 | 768 | 3720 | 6466 | 2418 | 1625 | 3772 | 1240 |
| 86D | 3237 |  |  | 3294 |  |  | 5919 |  |  |
| 86D | 6069 |  |  | 12384 |  |  |  |  |  |
